# Examining the benefits and harms of Alzheimer’s disease screening for family members of older adults: study protocol for a randomized controlled trial

**DOI:** 10.1186/s13063-019-4029-5

**Published:** 2020-02-19

**Authors:** Nicole R. Fowler, Katharine J. Head, Anthony J. Perkins, Sujuan Gao, Christopher M. Callahan, Tamilyn Bakas, Shelley D. Suarez, Malaz A. Boustani

**Affiliations:** 1 Department of Medicine, Indiana University School of Medicine, Indianapolis, IN 46202 USA; 2 Indiana University Center for Aging Research, Indianapolis, IN 46202 USA; 3 Regenstrief Institute, Inc., Indianapolis, IN 46202 USA; 4 Center for Health Innovation and Implementation Science, Indiana Clinical and Translational Science Institute, Indianapolis, IN 46202 USA; 5 Department of Communication Studies, Indiana University-Purdue University Indianapolis, Indianapolis, IN 46202 USA; 6 Department of Biostatistics, Indiana University School of Medicine & Richard M. Fairbanks School of Public Health, Indianapolis, IN 46202 USA; 7 Eskenazi Health, Indianapolis, IN 46202 USA; 8 College of Nursing, University of Cincinnati, Cincinnati, OH 45219 USA

**Keywords:** Alzheimer’s disease, Dementia, Screening, Caregivers, Family, Benefits, Harms

## Abstract

**Background:**

Multiple national expert panels have identified early detection of Alzheimer’s disease and related dementias (ADRD) as a national priority. However, the United States Preventive Services Task Force (USPSTF) does not currently support screening for ADRD in primary care given that the risks and benefits are unknown. The USPSTF stresses the need for research examining the impact of ADRD screening on family caregiver outcomes.

**Methods:**

The Caregiver Outcomes of Alzheimer’s Disease Screening (COADS) is a randomized controlled trial that will examine the potential benefits or harms of ADRD screening on family caregivers. It will also compare the effectiveness of two strategies for diagnostic evaluation and management after ADRD screening. COADS will enroll 1800 dyads who will be randomized into three groups (*n* = 600/group): the ‘Screening Only’ group will receive ADRD screening at baseline and disclosure of the screening results, with positive-screen participants receiving a list of local resources for diagnostic follow-up; the ‘Screening Plus’ group will receive ADRD screening at baseline coupled with disclosure of the screening results, with positive-screen participants referred to a dementia collaborative care program for diagnostic evaluation and potential care; and the control group will receive no screening. The COADS trial will measure the quality of life of the family member (the primary outcome) and family member mood, anxiety, preparedness and self-efficacy (the secondary outcomes) at baseline and at 6, 12, 18 and 24 months. Additionally, the trial will examine the congruence of depressive and anxiety symptoms between older adults and family members at 6, 12, 18 and 24 months and compare the effectiveness of two strategies for diagnostic evaluation and management after ADRD screening between the two groups randomized to screening (Screening Only versus Screening Plus).

**Discussion:**

We hypothesize that caregivers in the screening arms will express higher levels of health-related quality of life, lower depressive and anxiety symptoms, and better preparation for caregiving with higher self-efficacy at 24 months. Results from this study will directly inform the National Plan to Address Alzheimer’s Disease, the USPSTF and other organizations regarding ADRD screening and early detection policies.

**Trial registration:**

ClinicalTrials.gov, NCT03300180. Registered on 3 October.

## Introduction

There are currently 5.4 million adults with Alzheimer’s disease and related dementias (ADRD) and 11 million informal caregivers in the USA [1], potentially rising to 13.8 and 27 million, respectively by 2050 [2, 3]. Currently, at least 50% of patients with ADRD are undiagnosed and, among those who are diagnosed, only half of the patients or their families know of the diagnosis [4–7]. Furthermore, the diagnosis often occurs 2–5 years after the onset of symptoms [8–11]. Although the United States Preventive Services Task Force (USPSTF) recommendations do not currently support screening for ADRD in primary care [12], multiple national expert panels who represent a broad range of stakeholders have identified early detection of ADRD as a national priority. Specifically, the National Academy of Science, the National Plan to Address Alzheimer’s Disease, and the Affordable Care Act (via the Medicare Annual Wellness Visit (AWV)) all identify earlier detection of ADRD as a core aim for improving the quality of care for older adults [13–15].

Of those patients with ADRD living in the community, 75% are cared for by family caregivers [16–18] and treated in primary care settings [19–21]. Barriers that lead to a delay in diagnosis can potentially lead to poorer patient and family caregiver outcomes [5, 6, 8, 22–24]. For example, delayed diagnosis perpetuates the belief that changes in cognition are part of “normal aging”, which has been shown to aggravate caregivers’ stress, burden and sense of isolation [22, 24, 25]. Furthermore, family members may not notice their own changing role, leaving them vulnerable or unprepared to become a caregiver [22, 26]. The USPSTF found no studies linking ADRD screening to improved family caregiver outcomes and stressed the need for research in this area [12, 27].

Those recommending earlier detection of ADRD believe that screening will: 1) increase opportunities for earlier medical, social, and advance care planning interventions; 2) identify and name the early cognitive, functional, psychological or behavioral symptoms as abnormal aging; and 3) provide an opportunity for family members to learn about the syndrome, prepare for the caregiver role, and plan for future care needs [5, 28–31]. The USPSTF has explicitly acknowledged the potential importance of identifying early cognitive impairment for family members and caregivers [12]. Thus, proactive approaches to early ADRD detection may improve the quality of life for family members who may ultimately become caregivers for their loved ones [19, 20, 29, 32]. The Medicare AWV came into effect in 2011 and includes detection of cognitive impairment as one of the annual routine assessments, citing these putative benefits [15]. In 2014, ≤14% of eligible beneficiaries received the AWV [33]. Analyses of the impact of the AWV on cognitive detection or care found that the AWV is correlated with an increase in some measures of cognitive care, such as laboratory testing for reversible causes for cognitive impairment, but it does not appear to have a substantial impact on improving the recognition of undetected ADRD [33].

Those who do not recommend routine ADRD screening believe that, without effective treatments, the emotional and social costs of screening are too high [34, 35]. For example, in a survey of 576 family members, caregivers for individuals who had received an ADRD diagnosis reported more relationship strain and reduced social activities compared with caregivers for individuals who had no official diagnosis, independent of the level of patient impairment [36]. These results imply that receiving a diagnosis once symptoms have emerged may elevate the importance of the disease, making it a more prominent part of an individual’s role and identity and perhaps prompting caregivers to become overly focused on providing personal care to the exclusion of their own well-being.

In response to these gaps in the knowledge base, our research team is conducting a randomized controlled trial, the Caregiver Outcomes of Alzheimer’s Disease Screening (COADS) study, to examine if ADRD screening impacts family member quality of life, depression and anxiety while controlling for detection that may occur as part of routine primary care or the AWV. The primary outcome in COADS is caregivers’ health-related quality of life; secondary outcomes are caregiver depression, anxiety, preparedness, and caregiving self-efficacy. We hypothesize that family members randomized to one of two screening arms will express higher levels of health-related quality of life, lower rates of depressive and anxiety symptoms, and report themselves to be more prepared for caregiving with higher self-efficacy at 24 months as compared to the control group without screening. Secondarily, the study will also examine the congruence of depressive and anxiety symptoms between older adults and family members and compare the effectiveness of two strategies for diagnostic evaluation and management after Alzheimer’s disease screening between dyads randomized to the two screening groups (‘Screening Only’ versus ‘Screening Plus’). Results from this study will directly inform the National Plan to Address Alzheimer’s Disease, the USPSTF and other organizations regarding their ADRD screening and early detection policies.

## Methods

### Study design

**Fig. 1:**
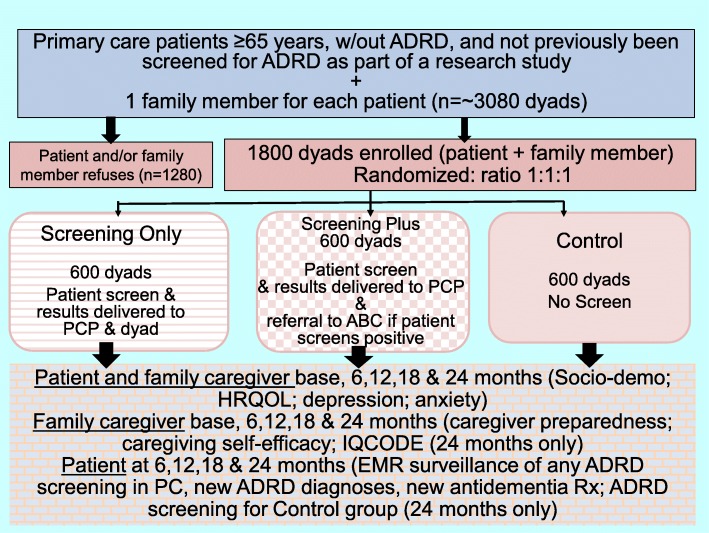
Recruitment, enrolment, and measures for the Caregiver Outcomes of Alzheimer’s Disease Screening (COADS) trial. ABC Aging Brain Care (Program), ADRD Alzheimer’s disease and related dementias, base baseline, EMR electronic medical record, HRQOL health-related quality of life, IQCODE Informant Questionnaire on Cognitive Decline in the Elderly, PC primary care, PCP primary care physician, Rx prescription

The COADS study is a multicenter, three-arm, randomized controlled trial. The aims of the study are to evaluate the impact of ADRD screening on family members’ quality of life, mood and anxiety. Additionally, the study will assess the impact of ADRD screening on family members’ caregiving preparedness and caregiving self-efficacy, compare the effectiveness of two strategies for diagnostic evaluation and management after ADRD screening, and examine congruence between patient–family member dyad outcomes among the three randomized groups. As shown in Fig. 1, the COADS trial will enroll 1800 patient–family member dyads from primary care clinics in Indiana, USA. These dyads will be randomized into three groups (*n* = 600 per group). Older adults in the first group will be screened for ADRD at baseline coupled with disclosure of the screening results to the dyad. If the patient screens positive, the dyad will receive a list of local resources for follow-up diagnostic care and disclosure of positive screening results to the patient’s primary care physician (the Screening Only group); older adults in the second group will be screened for ADRD at baseline coupled with disclosure of the screening results (as above) and, if they screen positive, a referral to a dementia collaborative care program (the Aging Brain Care (ABC) Program) for diagnostic evaluation and subsequent care if ADRD is diagnosed (the Screening Plus group). The ABC Program conducts diagnostic evaluations and delivers collaborative care management to patients diagnosed with ADRD and their family caregivers and is described in detail below. Older adults in the third group will not be screened at baseline and family members are given no information about cognition (the control group). In this group, we will observe, through surveillance of the electronic health record of the participant, any screening or incident diagnoses of ADRD that occur as part of routine care, including the Medicare AWV. At the end of follow-up (24 months), we will screen the older adults in this group and conduct an interview with the family member to detect possible cognitive impairment in the participant. The COADS trial will measure the primary and secondary outcomes at baseline and at 6, 12, 18 and 24 months. Consent, enrollment, data collection, and ADRD screening (if in a screening arm) will be obtained face-to-face in the primary care clinic or by telephone.

This study protocol has followed the Standard Protocol Items: Recommendations for Interventional Trials (SPIRIT) Guidelines (Additional file 1). The trial will be conducted and reported according to the reporting of pragmatic trials, an extension of the Consolidated Standards of Reporting Trials (CONSORT) statement. The study has been approved by the Institutional Review Board of Indiana University (IRB no. 1705649205). The COADS trial is registered with ClinicalTrials.gov (NCT03300180).

### Setting and study population

Patient–family member dyads for the COADS trial will be recruited from Eskenazi Health (EH) primary care sites and primary care sites affiliated with Indiana University Health (IUH). Recruitment will occur via the Indiana University Practice-Based Research Network, which covers research recruitment in all primary care practices affiliated with EH and IUH [37]. We have long-standing relationships with these sites which serve diverse populations of insured and uninsured patients throughout the Indianapolis metropolitan area and central Indiana. EH is the third largest safety net health system in the USA. We will recruit from primary care practices (all of which are Federally Qualified Health Centers) in Indianapolis that are associated with EH. We will have access to recruit from over 20 primary care practices in central Indiana that are affiliated with IUH, which includes more than 200 primary care providers. We will also use the Indiana University Practice-Based Research Network as our recruitment method [37]. Data generated by the Indiana University Practice-Based Research Network is collected and stored in the Indiana Network for Patient Care, which serves as the central Indiana Regional Health Information Exchange (IHIE) [38]. Our study team has long-standing relationships with the clinical sites participating in this trial, and has conducted numerous research activities and projects within their primary care practices [19, 39–50].

### Eligibility

The target population is dyads formed by: 1) an adult aged 65 or older without a diagnosis of ADRD; and 2) a family member or legal health care power of attorney whom the patient identifies as someone who would provide care for them if they needed it. Eligibility of patients will be established through screening the Indiana Network for Patient Care database and by assessments conducted by the research assistants face-to-face or via the telephone.

#### Inclusion criteria

The following inclusion criteria are employed for the patients: ≥65 years of age, have had at least one visit to the primary care practice within the last year, are able to provide informed consent, and can communicate in English. Family members serving as study partners must be ≥21 years of age, identified by the patient as the person most likely to provide care for them if needed, able to provide informed consent, able to communicate in English, and live within a 50 mile radius of the patient. Study partners who are not family members must meet family member inclusion criteria and be the legal health care power of attorney.

#### Exclusion criteria

Dyads will not be eligible if the patient has a diagnosis of ADRD (which we will identify via the Indiana Network for Patient Care by medical record review of the 10th Revision of the International Statistical Classification of Diseases and Related Health Problems (ICD-10) codes) [37], evidence of a prescription for cholinesterase inhibitors or memantine (medications used to treat patients with ADRD), has a serious mental illness (e.g., bipolar or schizophrenia as determined by ICD-10 code), is an established patient in the EH ABC Program, or is a permanent resident of a nursing facility. Dyads will also be excluded if the proposed study partner self-reports a serious mental illness (i.e., bipolar or schizophrenia as determined by ICD-10 code), has a diagnosis of ADRD determined by self-report, or is a nonfamily member who does not have legal health care power of attorney for the patient.

### Recruitment

Patients will be contacted first by letter to inquire about their interest in participating or approached in clinic at a visit to assess interest and eligibly. If they are interested, the team member then asks for the name and contact information of a family member and additionally requests permission to call the family member to assess if they are also willing to enroll. Given that not all patients will require the assistance of a caregiver during the study or have a person identified as a caregiver, we want to identify the person most likely to assume that role when patient needs arise.

Based on the literature and our own pilot data, we anticipate approaching approximately 3500 dyads to enroll 1800 dyads. Rolling enrollment will take place over 22 months with an average monthly enrollment of 80 dyads. The COADS trial will reduce loss to follow-up for longitudinal assessments by engaging the dyads every 6 months throughout the study (at 6, 12, 18 and 24 months), including keeping the assignment of research staff and participants consistent at each outcome assessment, sending reminder letters, and sending birthday cards signed by the study team. This strategy has produced <1% loss to follow-up in the pilot study.

### Randomization

The unit of randomization will be the patient–caregiver dyad. Dyads will be randomly assigned in 1:1:1 ratio to one of three groups (Screening Only or Screening Plus or control) and stratified by recruitment site to control for institutional effects and styles of different primary care providers.

Study statisticians will use a computer-generated randomization scheme (random block sizes of 3 or 6) stratified by the patient’s health care system (EH versus IUH) to assign dyads rather than providers or clinics to one of three groups to minimize the effects of unmeasured case mix differences and clinic-level clustering. Based on data from the COADS pilot and the literature, the risk for ‘spillover’ from having participating clinics treat both intervention and usual care patients is likely small [48].

### Description of the intervention

For both screening arms, we will use the Mini-Cog or the Memory Impairment Screen Telephone version (MIS-T) as our ADRD screening instrument [51, 52]. These instruments were selected to allow for flexibility in mode of screening and because both instruments demonstrate validity in primary care, are short and practical (4–5 min to complete with excellent inter-rater reliability), and have a positive likelihood ratio of 5 or higher [51–53]. The MIS-T has a total score from 0 point to 8 points. A cut-score ≤5 has 85% sensitivity and 86% specificity for dementia with a positive predictive value of 52% in a setting with a dementia prevalence of 15% [27, 51–53]. The Mini-Cog has a total score of 0–5 and takes 3–5 min to administer. A cut-score ≤2 has 76–99% sensitivity and 89–93% specificity for dementia with a positive predictive value of 32% [54–57]. The ADRD screening will be administered to the patient by the study research assistants after the dyad consents and is randomized into one of the two screening arms.

#### Screening Only

Dyads randomized to the Screening Only group will receive a letter that will include the results of the patient’s ADRD screening results provided in lay-language. For patients who score 6–8 on the MIS-T or ≥3 on the Mini-Cog, both the patient and family member will receive a letter indicating that they performed “within normal range”, but also will advise that if they have any concerns they should talk with their primary care physician. If the patient’s score is ≤5 on the MIS-T or ≤2 on the Mini-Cog, the letter states, “your score (or the patient’s score) on the test was lower than we would expect. This indicates that you may be having a problem with your thinking or memory”, and will encourage the patient and family member to discuss this with the patient’s primary care physician at the earliest possible time with the suggestion they ask about additional cognitive tests to identify possible causes. The primary care physicians of patients who screen positive in the Screening Only group will also receive a brief note of the patient’s screening test performance via the electronic health record system.

#### Screening Plus

Patients and family members randomized to the Screening Plus group receive the same letters described above, with additional electronic health record system notification to patient’s primary care physician upon a positive screen result. If the patient scores ≤5 on the MIS-T or ≤2 on the Mini-Cog, the letter will also indicate that the dyad will be receiving a call from the ABC Program along with the name and contact number for an ABC Program care coordinator (CC). All referrals from the study to the ABC Program will occur within 48 h of the positive screening result. Only dyads where the patient screens positive will be referred to the ABC Program. The patient’s primary care physician is also informed of the ABC Program referral and provided with information about the ABC Program, including the clinic’s providers and a general overview of what a follow-up visit would include for a dyad.

The ABC Program conducts diagnostic assessments and performs collaborative care management between the dyad, aging brain specialists, and the patient’s primary care physician. It includes a medical (MD, RN, and PharmD) and nonmedical (social workers, public health workers, and CC assistants) [46] workforce. This is a collaboration that is supported by evidence-based assessment tools (e.g., The Healthy Aging Brain Care Monitor) [58] and a technology platform (e.g., a mobile office and the care coordination electronic Medical Record Aging Brain Care (eMR-ABC) software) so care can be delivered wherever the person and their caregiver needs it [59]. Through its three phases, the program is targeted to comanage or support the practice behavior of primary care clinicians, enhance self-management skills of both the patient and caregiver, and maximize the coping behavior of the patient and the informal caregiver. By design, the previously tested program protocols lead to individualized and patient-centered profiles of actual interventions for individual patients and their informal caregivers [44, 50].

In phase 1 (initial assessment), a CC conducts a biopsychosocial needs assessment by telephone upon enrollment and schedules a clinic visit for a dyad for follow-up diagnostic testing. The telephone call includes a demographic and psychosocial interview focused on achieving problem identification using standardized assessment tools and eMR-ABC. Results from this assessment inform the clinic visit when neurological examination and neuropsychological testing are conducted. Also, at the clinic visit, the care team focuses on problem clarification and reviews the assessment findings, the medical record and medication lists, any diagnostic testing, any brain imaging results, and functional details of the assessment to determine the presence or absence of a likely dementia diagnosis, identifying any reversible and comorbid conditions. After the first visit and review of all findings from prior data, the care team creates an initial plan and identifies areas needing further assessment at a possible home visit.

In phase 2 (collaborative care plan development), the diagnostic report and individualized care plan is created and reviewed with the patient and the family caregivers. A summary is also sent to the patient’s primary care team. After phase 1 is completed, any urgent medical, behavioral, and psychological issues are addressed via consultation with specialists (e.g., geriatricians and neurologists) and/or the primary care physician as needed. The ABC team will map out a proposed care plan and schedule a home visit to review findings, discuss identified problems and propose a collaborative plan. During the home visit, the team reviews the identified problem list and seeks input from the dyad regarding the content of the list and the priority level of those problems, addressing first the issues felt to be most important by the patient. From this resulting consensus, the CC discusses a proposed individualized care plan, explains the diagnosis and natural history, implements appropriate care protocols, reviews, explains, and distributes the corresponding educational handouts for the dyad, and connects patients and family caregivers to in-home services and community resources as needed.

In phase 3 (follow-up phase), the CC team will continue to interact with the dyad face-to-face at their home or via telephone or email/mail. Interaction intensity will be dictated by the care plan, presenting needs, and circumstances. During these interactions, the CC will answer any questions generated from previous visits, collect dyad feedback, have the caregiver complete a brief assessment to identify need for specific care protocols, and facilitate the caregivers’ participation in an array of community services that are available in central Indiana. The CC will reconcile medications and review medication adherence at the home visits. Medication questions will be referred to the patient’s primary care physician. Throughout the duration of the follow-up phase, the team will continue to work with the dyad and the patient’s primary care physician to monitor, implement, and adjust the individualized care plan, as necessary.

### Description of the control

Patients in dyads who are randomized to the control arm (no screening) will not be screened at baseline. Similar to the design of cancer screening trials [60], this group will undergo active surveillance throughout the study via electronic health records to monitor any ADRD screening in primary care (routine or AWV), new diagnoses of ADRD, and new prescriptions for antidementia medications. At the 24-month outcome assessment, we will administer the MIS-T or Mini-Cog to the patient and the short form of the Informant Questionnaire on Cognitive Decline in the Elderly (short IQCODE) [61] to the family member to determine the patient’s cognitive status, and to differentiate which patients in this group may have impairment. This approach will allow comparison of family members in each of the three arms, by group, by patient screening status (positive versus negative), and by cognitive status (impaired versus not impaired).

COADS was designed with these three conditions, two of which include pathways for diagnostic assessment and care, for two main reasons: 1) to ensure that postscreening evaluations and care would not confound the effects of testing the impact of screening overall; and 2) to confirm that the evaluation of an ADRD screening program on outcomes should be consistent with previous screening studies in other conditions that found that disconnecting screening from a subsequent diagnostic process and care program reduces the impact of screening on the burden of the target condition [62].

### Primary and secondary outcome measures

The primary outcome measure is family member health-related quality of life. Secondary measures include family member depressive and anxiety symptoms, caregiving preparedness, caregiving self-efficacy, and patient depressive and anxiety symptoms. Primary and secondary outcome measures will be assessed at baseline and at 6, 12, 18 and 24 months by blinded research assistants.

#### Health-related quality of life

We will use the Medical Outcomes Study Short Form Health Survey 36 (SF-36) to determine the family members’ and patients’ health-related quality of life at each time point [63]. The SF-36 is a general population instrument that measures health-related quality of life and mental, physical and social functioning [64, 65]. It includes one multi-item scale that assesses the following eight health concepts: physical functioning, role-physical, bodily pain, general health, vitality, social functioning, role-emotional and mental health. These concepts are aggregated into a physical component summary (PCS) and a mental component summary (MCS). Changes that differ between groups by ≥2 points on a scale of 0 to 100 have been shown to be clinically or socially meaningful. Higher SF-36 scores indicate better health functioning [64]. The SF-36 is psychometrically sound and has been used extensively in older adults and caregivers of older adults with and without ADRD [66, 67].

#### Depressive and anxiety symptoms

We will use the Patient Health Questionnaire 9 (PHQ-9) [68, 69] and the Generalized Anxiety Disorder Scale (GAD-7) [70, 71] to determine the impact of ADRD screening on family members’ and patients’ mood and anxiety at each time point. The PHQ-9 is a nine-item depression scale with a total score from 0 to 27, and the GAD-7 is a seven-item anxiety scale with a total score from 0 to 21. Both of these scales are derived from the Patient Health Questionnaire, have good internal consistency, test–retest reliability, as well as convergent, construct, criterion, procedural, and factorial validity for the diagnosis of major depression and generalized anxiety disorder [69, 71]. In our previous studies conducted in primary care settings, the mean PHQ-9 scores ranged from 3.8 (SD = 5.1) to 4.4 (SD = 5.6), and the mean GAD-7 scores ranged from 2.7 (SD = 3.2) to 3.2 (SD = 3.5) [66]. In a study conducted by our group, the PHQ-9 was shown to be a valid measure of stressful events, both related and unrelated to caregiving [72]. Each of the above measures (SF-36, PHQ-9, and GAD-7) has been shown to be sensitive to change over time [66, 69–71].

#### Caregiver preparedness

Caregiver preparedness refers to how ready family members perceive they are for the tasks and demands of caregiving such as providing physical care and emotional support to the patient or dealing with the stress of caregiving if they were to experience a transition in role [73, 74]. Family members who report a high level of preparedness for caregiving have been shown to experience less worry and decreased levels of depression [75, 76]. In contrast, family members who perceive themselves as being inadequately prepared are prone to greater levels of burden [77]. Preparedness for caregiving is associated with generally lower levels of caregiver strain and has a broad effect on multiple indicators of quality of life and total mood disturbance [75–77]. Although previous claims in the literature have stated that earlier identification of ADRD through screening can help prepare caregivers, no data exist to support this claim [12]. Our trial will test if earlier identification of ADRD through screening impacts caregiver preparedness.

We will use the Preparedness for Caregiving Scale [77] to measure the impact of ADRD screening on family member preparedness. The Preparedness for Caregiving Scale consists of eight items that asks family members how well prepared they believe they are for multiple domains of caregiving. Responses are rated on a five-point scale with scores ranging from 0 (not at all prepared) to 4 (very well prepared). The scale is scored by calculating the mean of all items answered with a score range of 0 to 4. The higher the score, the more prepared the caregiver feels for caregiving; the lower the score, the less prepared the caregiver feels. Internal consistency of this scale has been reported as excellent with alpha scores ranging from 0.88 to 0.93 [75, 78].

#### Caregiver self-efficacy

Research suggests that self-efficacy may be an important construct to understanding how family members of older adults cope with the role transition to a caregiver and the subsequent challenges and demands of providing care [79]. Among family caregivers of older adults with ADRD, Aneshensel and colleagues found that caregivers’ self-efficacy, particularly their sense of mastery and control about their caregiving role, had a direct and positive effect on reducing caregiver depression over time [80, 81].

The COADS study utilizes the Revised Scale for Caregiving Self Efficacy [82] to measure the impact of ADRD screening on caregiver self-efficacy. The scale is a 15-item scale that evaluates family members’ capacity in relation to the caregiving role, more specifically with regard to obtaining respite from caregiving by the involvement of family and friends (e.g., asking a friend or family member to stay with the patient for a day to take a break). The scale also inquires about family members’ perceived control over disturbing thoughts that may arise about the caregiver role (e.g., unfairness of having to manage this caregiving situation) and their response to the relative’s disruptive behaviors (e.g., responding without raising your voice when your relative interrupts your activities repeatedly). Respondents rate their degree of self-efficacy on a scale from 0 (absolutely incapable) to 100 (fully capable). Pearson correlation coefficients between the overall scale and the three specific dimensions ranged from 0.66 to 0.73 (*P* < 0.001). The alpha coefficient for the overall scale was 0.86 [79–82].

#### Other measures

The COADS study additionally collects data from patients’ electronic health records to assess if and when they undergo any ADRD screening (outside of the COADS trial) as part of their routine primary care, an AWV, or specialty care. Data generated as part of an encounter by a provider who practices within EH and IUH are stored in the Indiana Network for Patient Care, which serves as the central IHIE and is available to clinical researchers to assess health events and utilization [37]. For dyads who are not randomized to screening at baseline (control group), cognition will be assessed at the end of the study at the 24-month assessment. Patients will be screened with the MIS-T or Mini-Cog, and the IQCODE is administered to all family members. The IQCODE screening tool is a short questionnaire designed to assess cognitive decline and dementia in older adults, informed by a relative; the 16 questionnaire items are summed and divided by 16. Using IQCODE cut-offs commonly employed in clinical practice (3.3–3.6) the sensitivity and specificity of IQCODE for the diagnosis of dementia is generally above 75% in community-dwelling older adults [83]. Information obtained from the MIS-T, Mini-Cog, and IQCODE results will allow for comparison of family member responses between the three groups sorted by the patients’ cognitive status.

### Data monitoring

The Data Safety Monitoring Plan (DSMP) for this trial will be monitored by the Principal Investigator and a five-member Data Safety and Monitoring Board (DSMB). The DSMB charter contains a detailed list of the DSMB responsibilities. The DSMB will act in an advisory capacity to the Institutional Review Board and National Institute on Aging Program Official to monitor participant safety, evaluate the progress of the study, and review procedures for data management and analysis, maintaining the confidentiality of data, and the quality of data collection.

Potential adverse events that will be monitored in the COADS study include patient or family member death, any event that is life threatening or places the participant at immediate risk of death, requires or prolongs hospitalization, causes persistent or significant disability or incapacity, or another condition which investigators judge to represent significant hazards. Developments in this realm will be monitored on an ongoing basis by the COADS research manager and discussed weekly among the research team. All adverse events and unanticipated problems will be reported to the study Principal Investigator within 24 h of discovery. If unanticipated serious adverse events occur (i.e., not listed in the DSMP) and are related to the intervention they will be reported within 48 h.

### Data collection

Data for the COADS trial will be collected at baseline and at 6, 12, 18 and 24 months. Following the confirmation of eligibility and completion of informed consent, all family members and patients in each of the three arms will complete the baseline assessment, which includes the SF-36, PHQ-9 and GAD-7. Family members at baseline will also complete the Caregiver Preparedness Scale, Caregiving Self-efficacy Scale, and the Oberst Caregiving Burden Scale (at baseline only) [84, 85]. Data will be collected face-to-face at the clinic or via telephone; data will also be obtained via electronic health record review. Research staff who administer follow-up assessments will be blinded to dyad intervention status and will be trained to read all questionnaires verbatim, without commentary. In addition to the primary and secondary measures, the study will collect social and demographic data on all patients and family members, including age, sex and race. The assessments will also record the relationship of the family member to the patient, the frequency and type of contact with the patient, geographic distance from the patient, education level, annual income, self-reported health status, and whether or not they have knowledge as to whether the patient has ever been screened for ADRD as part of routine primary care, an AWV, or a community screening event. Additional patient descriptive data will be obtained from the recruitment site databases and the Indiana Network for Patient Care including, but not limited to, comorbidities. All survey data will be entered into a database using Research Electronic Data Capture (REDCap), a secure web-based application available through our Clinical and Translational Science Institute.

### Timeline

The recruitment of patients began on 15 October 2018 and is expected to be finalized by August 2020. All data collected from recurring outcome assessments is expected to be collected by 2022. The data analysis, writing of scientific manuscripts, and submissions to peer-reviewed scientific journals will occur from 2020 to 2023.

### Analysis plan

#### Comparison of baseline characteristics

The first examination will analyze univariate distributions of continuous variables to detect any potential violations of assumptions to our planned parametric methods of analysis. Variables will be transformed as needed to ensure normal distribution assumptions are met. Nonparametric methods will be used if transformations are inadequate. Demographic characteristics will be compared among the groups to evaluate whether the randomization effectively balances the dyads. We will use Chi-squared tests or Fisher’s exact tests to compare the frequencies of categorical variables. Analysis of variance (ANOVA) or its nonparametric alternative, the Wilcoxon rank sum test, will be used to compare the distribution of continuous variables among the groups. All analyses will be conducted using SAS 9.4 (SAS Institute, Carey, NC, USA).

#### Comparison of outcomes

Since our randomized controlled trial is randomizing patients in dyads to ADRD screening or no screening, our planned analyses will utilize multilevel mixed-effects models to capture repeated measures from both patients and family members. Multilevel mixed-effects models will be used to examine differences in SF-36 scores, PHQ-9 or GAD-7 for both patients and family members using dyadic analytic approaches comparing dyads in the two screening groups (Screening Only and Screening Plus) to those in the no-screening (control) group. Repeated SF-36, PHQ-9 or GAD-7 scores from both patients and family members will be included as the outcome variables with participant type (patient or family member), group (Screening Only and Screening Plus versus control), time, and interaction between groups and time as independent variables [86]. We will use a multilevel variance–covariance matrix in the mixed effects models to account for two sources of potential correlations: 1) correlations from measures obtained from the same individual over time (an autoregressive correlation will be used); and 2) correlations within a dyad between a patient and his/her family member (a compound symmetry structure will be used for the intra-dyad correlation). Parameter estimation and hypothesis tests for the mixed-effects models will be conducted using the maximum likelihood approach that provides robust estimation under the missing at random mechanisms [87]. A significant interaction between group and time would indicate differences in changes of SF-36, PHQ-9 or GAD-7 over time between the screening groups and the no-screening group. In the absence of significant interactions, significant main group effects would suggest differences in the outcome between groups at all follow-up time points. We will use a linear contrast for SF-36, PHQ-9 or GAD-7 from family members to compare these outcomes in the combined screening groups (Screening Only and Screening Plus) versus the no-screening group (control) at 24 months. We will also include additional covariates in the mixed-effects models to determine whether family member characteristics (e.g., relationship to patient, frequency or types of contact) and knowledge of screening are associated with the outcome measures.

The study will also assess the impact of ADRD screening on family members’ caregiving preparedness and caregiving self-efficacy using mixed-effects models with the Caregiver Preparedness Scale and the Revised Scale for Caregiver Self-Efficacy scores collected at baseline and at 6, 12, 18 and 24 months as the outcome variables, and group (Screening Only and Screening Plus versus control), time, and interaction between group and time as independent variables. Linear contrasts will be used to compare preparedness and caregiver self-efficacy scores in the combined screening groups versus the no-screening group at 24 months. We will also evaluate potential interactions between patients/family member characteristics and variables associated with increased level of caregiver preparedness and self-efficacy over time.

To measure the impact and compare strategies for evaluation and treatment postscreening, this study will compare quality-of-life measures, caregiver preparedness, caregiving self-efficacy, and depression and anxiety symptoms as reported from family members in the Screening Only group to those in the Screening Plus group. The screening groups differ by the amount of postscreening contact, therefore possible outcome differences between these two groups will be analyzed using multilevel mixed-effects models, similar to the approach described in detail above. Separate mixed-effects models with SF-36, Caregiver Preparedness Scale, Revised Scale for Caregiver Self-Efficacy, PHQ-9 or GAD-7 scores collected at baseline and at 6, 12, 18 and 24 months from family members will be used as the outcome variables. Screening group (Screening Only versus Screening Plus), time, and interaction between group and time will be used as independent variables. Linear contrasts will be used to compare all scale scores between the two screening groups at 24 months.

#### Sensitivity analyses for the impact of refusals and other sources of missing data

We will compare patient and family member characteristics between 1) those who complete at least one or more follow-up assessment(s) after baseline and 2) those who did not complete any assessment beyond baseline due to refusal or other reasons. Significant variables detected from these comparisons will be included in the mixed-effects models for the primary and secondary outcomes as covariates to control for potential bias from those who did not complete any follow-up outcomes. Under the missing at random assumption, results from the mixed-effects models will remain unbiased if the variables contributing to the missing data are included as covariates in the models. We will also perform additional sensitivity analyses to examine whether our analyses are impacted by the missing at random assumption using the selection model approach under an informative missing mechanism [87].

#### Sample size and statistical power analysis

For the models examining the impact of ADRD screening on caregivers’ quality of life, mood, anxiety, preparedness and self-efficacy, we assume a base correlation of 0.2 and a decay rate of 0.8 in a linear exponent autoregressive correlation structure for repeated measures and a continuous time response. We will need to have 540 dyads per group to have complete data at 24 months in order to achieve 82.6% power to detect a group by time interaction, with an effect size of 0.24 SD where there are higher SF-36, lower PHQ-9, lower GAD-7, higher caregiver preparedness and self-efficacy scores in the combined screening groups (Screening Only and Screening Plus) at 24 months compared to family members in the no-screening (control) group at 24 months. Therefore, allowing a loss to follow-up rate of 10% over the 24 months, the study plans to enroll 600 dyads per group into this study. In order to compare the effectiveness of the two strategies for diagnostic evaluation and management after ADRD screening, similar assumptions are employed regarding the correlation structure for repeated measures and, with a 10% attrition rate, there will be an 83.4% power to detect a significant group and time interaction, with an effect size of 0.28 SD when comparing family members randomized to the Screening Plus group to those in the Screening Only group at 24 months. Power estimation was conducted using the GLMPower procedure in SAS.

## Discussion

Although multiple national expert panels who represent a broad range of stakeholders, including the National Academy of Science, the National Plan to Address Alzheimer’s Disease, and the Affordable Care Act, through the AWV, have identified early detection of ADRD as a national priority [12, 15], the USPSTF recommendations do not currently support screening for ADRD in primary care [12]. The COADS trial will be the first to examine the potential benefits or harms of ADRD screening specifically on caregivers’ quality of life, mood, anxiety, preparedness, and self-efficacy. It will also compare the effectiveness of two strategies for diagnostic evaluation and management after ADRD screening.

The COADS trial will use an innovative design that is common in cancer screening trials to create three groups to compare family member outcomes as a result of screening and postscreening strategies and to analyze individual outcomes and congruence between patient–family member dyad outcomes. It will randomize 1800 dyads into three groups: a Screening Only group, a Screening Plus group, and a control group. This randomized design will allow us to investigate the relationship between patient exposure to ADRD screening and family member outcomes as a result of the exposure, with minimum confounding bias. This study design will also produce the necessary data to examine if the risks and benefits of screening experienced by patients align or diverge with those of family members.

Having two different postscreening approaches (routine primary care versus dementia collaborative care) and conducting real-world surveillance of any screening in the no-screen group will also separate the impact of the screening event from the follow-up care and allow us to test organized screening (Screening Only and Screening Plus groups) versus usual care (no-screen control group). This design will allow us to test the effect of screening on its own and to test two different strategies for implementing a postscreening policy. Although coupling screening with diagnostic services and care is consistent with the World Health Organization’s Principles and Practices of Screening for Disease [88], we recognize that dementia collaborative care is not available throughout the USA and that it may confound the effects of screening versus no screening if those who screen positive are only referred to a collaborative care program. This innovative aspect of our design will allow us to test if collaborative care as part of screening is an essential part of any ADRD screening program and if disconnecting screening from a subsequent diagnostic process and care program reduces the impact of screening on the burden of the target condition.

The present study has some limitations. The first and most significant threat to the success of the COADS trial may be recruitment of both members of the patient–family member dyad. Some older adults will have no family that can participate, and some family members may be unwilling to view themselves as potential future caregivers. The second limitation is that some patients eligible for this study and randomized to the no-screen (control) group may be screened for ADRD as part of routine primary care. A third limitation is that dyads who are in the Screening Plus group may refuse to seek follow-up from the ABC Program if they screen positive. To assess and prepare for these possible limitations, we have: 1) conducted a large pilot to assess the number of potential dyads who both agree to consent; 2) piloted the protocol and measures; 3) collected pilot data from dyads to assess knowledge about the patient’s screening status either as part of research or during routine primary care; 4) conducted a feasibility study using data from our regional health exchange to measure counts of screening or AWVs; and 5) collected pilot data of how many patients refuse diagnostic assessment following a positive screen and adjusted our sample size to account for this. In our pilot study, 82% of patient–family members dyads approached for the COADS pilot agreed to participate. And, although screening in routine primary care may confound the impact of screening on family members, the prevalence of the AWV is still small and our design is sound and pragmatic as it mirrors what is likely to occur in primary care. As previously noted, in 2014 only 14% of eligible Medicare beneficiaries received an AWV [33]. In preparation for this study, we found that among a sample of ~44,000 Medicare beneficiaries in the EH and IUH population <8% had a documented AWV in 2016. Lastly, although randomized trials are the gold standard for evidence-based decision making in medicine, they can have important limitations as a basis for screening policies. First, screening policy development demands information about long-term benefits and harms because these policies generally pertain to interventions conducted over an individual’s healthy lifetime. We have extended our outcome to 24 months, but the long-term outcomes generated by a typical population-based screening program may not be fully realized for 5–8 years [89, 60].

In summary, as the number of patients with ADRD increases, so will the number of family caregivers. Although the need for early detection of ADRD is frequently debated as important care for older adults, this includes the need to support their family members to prepare for caregiving needs. The effects of routine screening on reducing the burden of ADRD and improving outcomes for family caregivers are unknown. The COADS trial will be the first of its kind to test the impact of ADRD screening and postscreening care pathways on family members using longitudinal data that is collected synchronously with patient outcome data. Following dyads after screening in diverse primary care settings including a large segment of minority, underprivileged, and rural populations will directly inform the National Plan to Address Alzheimer’s Disease, the USPSTF and other organizations regarding their ADRD screening and early detection policies.

### Trial status

This study received ethical approval from the Indiana University Institutional Review Board on 20 July 2017. The first dyad was enrolled on 15 October 2018. To date, 868 dyads (1736 participants) have been randomized so far. We are still recruiting patients and plan to close the recruitment at the end of 2020.

## Supplementary information

**Additional file 1.** SPIRIT 2013 checklist: recommended items to address in a clinical trial protocol and related documents.

## Data Availability

Not applicable.

## Abbreviations

ABC Program: Aging Brain Care Program
ADRD: Alzheimer’s disease and related dementias
ANOVA: Analysis of variance
AWV: Annual Wellness Visit
CC: Care coordinator
COADS: Caregiver Outcomes of Alzheimer’s Disease Screening
CONSORT: Consolidated Standards of Reporting Trials
DSMB: Data Safety and Monitoring Board
DSMP: Data Safety Monitoring Plan
EH: Eskenazi Health
GAD-7: Generalized Anxiety Disorder Scale
IHIE: Indiana Regional Health Information Exchange
IQCODE: Informant Questionnaire on Cognitive Decline in the Elderly
IUH: Indiana University Health
MCS: Mental component summary
MIS-T: Memory Impairment Screen Telephone version
PCS: Physical component summary
PHQ-9: Patient Health Questionnaire 9
REDCap: Research Electronic Data Capture
SF-36: Medical Outcomes Study Short Form Health Survey 36
SPIRIT: Standard Protocol Items: Recommendations for Interventional Trials
USPSTF: United States Preventive Services Task Force
